# Switching to dual therapy with darunavir/ritonavir and etravirine: a simplification strategy

**DOI:** 10.1186/1758-2652-13-S4-P51

**Published:** 2010-11-08

**Authors:** M Tyrer, L Swaden, NJ Marshall, M Johnson

**Affiliations:** 1 Royal Free Hospital, London, UK; 2 Royal Free Hospital NHS Trust, London, UK

## Background

Long term maintenance with NRTI-sparing regimens may be preferable for patients with NRTI toxicities, and may offer potential cost savings. Dual ART with once daily darunavir/r and etravirine may be preferable to previous PI/r/NNRTI combinations due to its theoretical higher genetic barrier to resistance and good PK profile. We looked at the use of this regimen within our HIV cohort.

## Methods

Patients prescribed dual ART with darunavir/ritonavir 800mg/100mg QD with etravirine 400mg/day (DRV/r/ETR) until January 2010 were identified by our virtual clinic database. Reason for switch, HIV resistance, viral outcomes were identified.

## Results

21 patients were switched to DRV/r/ETR with median time on regimen of 51.5 weeks (IQR 33-69 wks). 85% (18/21) where given ETR 400mg QD. 62% (13/21) switched from dual PI/r regimens, 10 combined with efavirenz or nevirapine. 28% (6/21) switched from conventional cART (2 NRTI + PI/r or NNRTI). Patients had a median exposure to 9 ARV drugs prior to switch (IQR 4-11), with 90% (19/21) having previous NNRTI exposure, 7 of which had CNS toxicity with efavirenz. At switch, 57% (12/21) had no previous resistance, 19% (4/21) NRTI mutations only, and 19% (4/21) had NNRTI mutations (K103N (2), Y181C combined with NRTI K65R, M184V mutations (1), prior NNRTI failure (2)). 90% (19/21) had VL<50cps/ml at switch, with 95% (20/21) achieving/maintaining VL<50cps/ml on regimen. Four patients discontinued the regimen, 2 switching to darunavir/r monotherapy, one switching to kivexa/darunavir/r due to non-adherence, and one switching back to previous regimen after 4 weeks. One patient was lost to follow up. Median virological follow of patients remaining on therapy was up 40.8 wks (IQR 32-58 wks). Median CD4 change for the 17/21 who remained on therapy was +101 cells/mm3 (IQR -50-138) with median 39 wks follow up (IQR 31-58 wks). Indications for switch were desire for simplification (9) (typically from dual PI), and need for NRTI-sparing regimen (12), including previous renal toxicity with tenofovir (4), lipoatrophy (7), peripheral neuropathy (1) and lactic acidosis (2).

## Conclusions

For patients with VL<50cps/ml, simplification to dual therapy with darunavir/r 800/100mg QD plus etravirine 400mg QD maintains viral suppression and immune reconstitution.

